# *Candida auris*: a review of global epidemiology, multidrug resistance, and infection control in healthcare-associated outbreaks

**DOI:** 10.3389/fcimb.2026.1873741

**Published:** 2026-07-10

**Authors:** Xuewei Du, Li Li

**Affiliations:** Department of Public Health, Affiliated Hospital of Inner Mongolia Medical University, Hohhot, Inner Mongolia, China

**Keywords:** antifungal resistance mechanisms, antifungal stewardship, biofilm formation, *Candidozyma auris*, clade diversity, climate change and fungal emergence, diagnostic challenges, emerging fungal pathogen

## Abstract

*Candida auris*, designated a critical-priority fungal pathogen by the World Health Organization, poses a growing threat to healthcare systems worldwide. Analysis of 80 peer-reviewed studies reveals five principal findings: (1) *C. auris* exhibits clade-dependent geographic distribution and resistance profiles—Clade I (South Asia) and Clade III show fluconazole resistance exceeding 90%, whereas Clade IV exhibits 44% resistance; echinocandin resistance varies significantly by clade and no resistance was detected in certain clades; amphotericin B resistance varies from no detected resistance to 46% depending on clade; and pan-resistant strains have emerged; (2) biofilm-associated *C. auris* cells (sessile cells) exhibit MBECs 2- to 4,119-fold higher than planktonic MICs, representing a major driver of persistent colonization; (3) novel antifungal agents, including ibrexafungerp, manogepix (the active moiety of fosmanogepix), and rezafungin, demonstrate promising *in vitro* activity against *C. auris*, with manogepix showing the highest overall antibiofilm activity (geometric mean MBEC of 5.9 μg/mL) and ibrexafungerp demonstrating superior activity against Clade IV biofilms; (4) a tiered infection prevention and control (IPC) framework—integrating universal screening, contact precautions, and environmental decontamination—has been associated with reduced transmission rates in outbreak settings; and (5) climate change and global warming may have contributed to the emergence of *C. auris* through thermal adaptation of environmental fungal species. These findings indicate that effective mitigation of the *C. auris* threat requires integrated surveillance, susceptibility-guided therapy accounting for both planktonic and biofilm activity, and resilient healthcare systems adapted to the clade-specific epidemiology of this pathogen.

## Graphical Abstract

**Figure:**
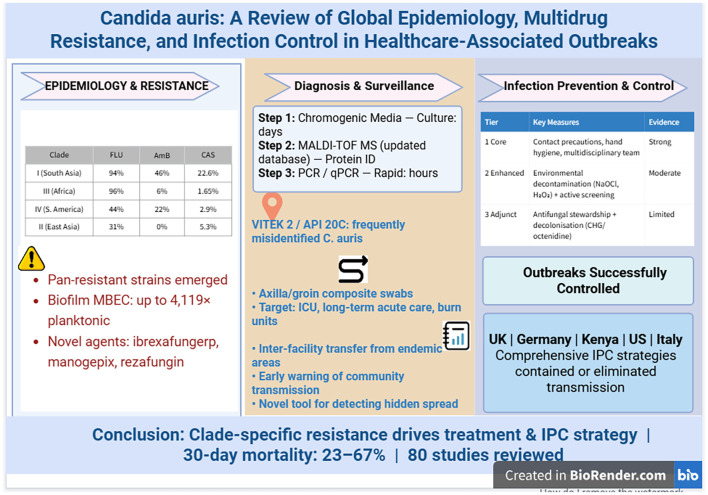
GA visual overview of the key themes addressed in this review on *Candida auris*. The figure integrates four core domains: (1) Global Epidemiology — depicting the geographic distribution of the five confirmed clades (I–V) and the proposed Clade VI, with emphasis on clade-specific transmission dynamics and the amplifying effect of healthcare system disruptions (e.g., COVID-19); (2) Antifungal Resistance — summarizing clade-dependent resistance profiles to fluconazole, amphotericin B, and echinocandins, the emergence of pan-resistant strains, biofilm-associated tolerance (MBECs up to 4,119-fold higher than planktonic MICs), and the *in vitro* promise of novel agents (ibrexafungerp, manogepix, rezafungin) against both planktonic and sessile cells; (3) Diagnosis and Surveillance — highlighting MALDI-TOF MS identification, molecular confirmation, and active screening of high-risk populations; and (4) Infection Prevention and Control — presenting the tiered IPC framework (screening, contact precautions, environmental decontamination, and antifungal stewardship) as an integrated strategy to contain transmission.

## 1 Introduction

*Candida auris* is among the most challenging emerging fungal pathogens of the 21st century. Since its first description in 2009 from a strain initially isolated from a patient’s external ear canal in Japan in 2008, this multidrug-resistant fungus has swept across the globe within just over a decade, being designated one of the “critical priority” fungal pathogens by the World Health Organization (WHO) and classified as an “urgent threat” by the United States Centers for Disease Control and Prevention (CDC) (World Health Organization, 2022; Chowdhary et al., 2023; Kim et al., 2024). Unlike the commonly encountered *Candida albicans* in clinical settings, *C. auris* possesses three core attributes that render it an exceptionally successful nosocomial pathogen: extensive intrinsic and acquired antimicrobial resistance, highly efficient transmission capability within healthcare facilities, and formidable diagnostic challenges (Forsberg et al., 2019; Chowdhary et al., 2023).

First, the threat posed by *C. auris* is manifested in its concerning resistance profiles. According to a recent systematic review of 50 studies analyzing 1,031 isolates (da Silva et al., 2025), fluconazole resistance rates exceed 90% in the predominant Clade I (94%) and Clade III (96%), while Clade IV shows a lower rate of 44%. A substantial proportion of strains exhibit reduced susceptibility to amphotericin B and echinocandin agents, and pan-resistant strains demonstrating resistance to all three major classes of antifungal drugs have emerged (Forsberg et al., 2019; Kim et al., 2024). This degree of resistance is exceedingly rare within the *Candida* genus and severely constrains therapeutic options (Arendrup and Patterson, 2017). Second, *C. auris* possesses an extraordinary capacity for prolonged, high-level colonization on patient skin, which serves as the foundation for its transmission within healthcare facilities (Chowdhary et al., 2025; Eix and Nett, 2025). The organism can survive on dry inanimate surfaces for weeks and tolerate many conventional disinfectants, thereby establishing persistent contamination reservoirs within hospitals. Third, *C. auris* is frequently misidentified as other *Candida* species by conventional biochemical identification systems, leading to diagnostic delays and delayed outbreak detection (Dewaele et al., 2019; Iguchi et al., 2019). The economic burden is also substantial: the cost of treating all invasive fungal infections in the United States alone exceeded $11 billion in 2019, and individual *C. auris* outbreaks have been associated with substantial incremental costs due to prolonged hospitalizations, isolation requirements, and environmental decontamination (Chowdhary et al., 2025).

In terms of disease burden, the overall mortality rate of *C. auris* bloodstream infections ranges from 30% to 60%, with 30-day mortality reaching 23% to 67%, varying by clade, geographic region, and patient population (Forsberg et al., 2019; Kim et al., 2024). Associated hospitalizations are significantly prolonged, with median lengths of stay ranging from 46 to 68 days, resulting in substantial consumption of healthcare resources (Kim et al., 2024). In recent years, the COVID-19 pandemic has further exacerbated the *C. auris* threat, with multiple countries reporting significant increases in *C. auris* infections or outbreaks among COVID-19 patients (Najeeb et al., 2022; Rahman et al., 2024).

Confronting this continuously evolving global health threat, in-depth epidemiological understanding and resolute execution of infection control measures constitute the two central pillars for containing its spread. However, existing reviews have predominantly discussed molecular resistance mechanisms, pathogen biology in isolation, and clinical therapeutics in parallel, resulting in diffuse foci and a lack of unifying themes (Chowdhary et al., 2023). This review, with “epidemiology and infection control” as its central theme, systematically synthesizes the global epidemiological characteristics, antifungal resistance epidemiology, diagnostic and surveillance technologies, and the latest advances in infection prevention and control strategies for *C. auris*, aiming to provide a focused, evidence-based reference for clinical and public health practitioners. It is noteworthy that *C. auris* was recently reclassified into the genus Candidozyma based on phylogenomic analyses (da Silva et al., 2025); both nomenclatures are used in this review to maintain consistency with the cited literature.

## 2 Methods

**Figure 1:**
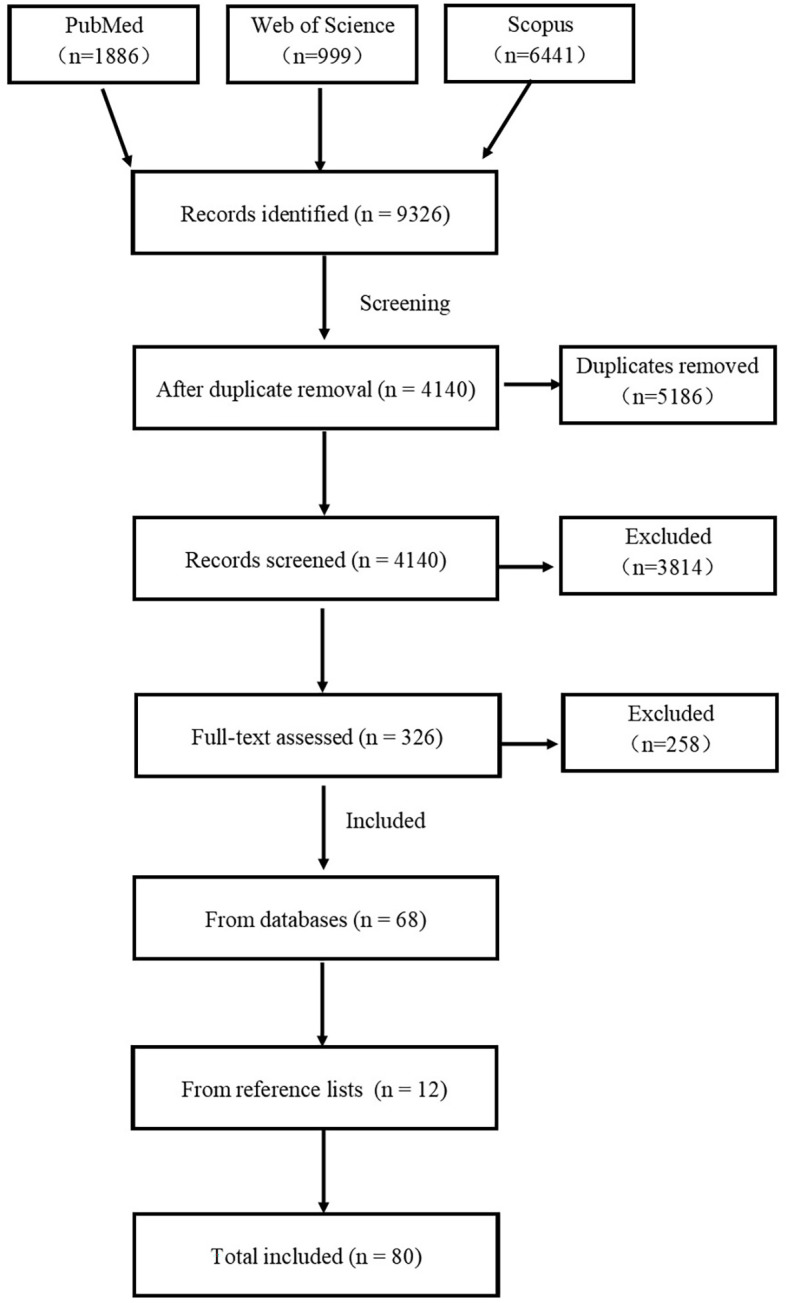
Key data summary. **(A)** antifungal resistance rates by clade; **(B)** global clade distribution; **(C)** planktonic MIC vs biofilm MBEC; **(D)** tiered IPC framework.

Search Strategy and Study Selection. For this narrative review, we systematically searched PubMed, Web of Science, and Scopus databases for English-language articles published from inception to March 2026 (search date: 21 March 2026) using the terms “*Candida auris*” OR “*Candidozyma auris*” combined with “epidemiology”, “antifungal resistance”, “infection control”, “diagnosis”, or “treatment”. Studies were included if they: (i) reported original data on *C. auris* epidemiology, antifungal resistance, diagnosis, or infection control; (ii) were published in peer-reviewed journals; and (iii) were available in English. Case reports, editorials, letters without original data, and non-English-language articles were excluded. The initial database searches yielded a total of 9,326 records (PubMed: 1,886; Web of Science: 999; Scopus: 6,441). After removal of 5,186 duplicate records, 4,140 unique records were screened by title and abstract by two independent authors, with discrepancies resolved by consensus; 3,814 records were excluded at this stage. The remaining 326 full-text articles were assessed for eligibility, of which 258 were excluded. This yielded 68 studies from the database searches; an additional 12 relevant publications were identified by screening the reference lists of included articles, bringing the total to 80 studies included in this review. The complete literature screening process is illustrated in **Figure 1**.

## 3 Infection prevention and control strategies

### 3.1 Multidisciplinary collaborative prevention and control system

The persistent colonization and efficient transmission characteristics of *C. auris* in healthcare environments make rigorous infection prevention and control measures the central means of containing outbreaks (Welsh et al., 2021; Solak Grassie et al., 2025). The success of prevention and control efforts depends critically on the rapid identification of colonized or infected patients and the immediate implementation of a multi-layered, coordinated comprehensive strategy, which requires a hospital-wide response transcending traditional infection control paradigms.

Establishing an efficient multidisciplinary response team is the first essential step. Expert consensus emphasizes that clinical departments, nursing teams, hospital infection control units, clinical microbiology laboratories, environmental cleaning personnel, and hospital administration must collaborate closely (Aldejohann et al., 2022; Jones et al., 2024). This collaborative model has proven crucial when responding to first outbreaks or imported cases in Germany, the UK, and Mexico, ensuring that measures from case identification and isolation to environmental terminal disinfection can be executed rapidly and consistently (Aldejohann et al., 2022; Hinrichs et al., 2022; la Garza et al., 2024). The clinical microbiology laboratory plays a “sentinel” role in this system, as its rapid and accurate identification capability forms the foundation for triggering all subsequent prevention and control measures. Once *C. auris* is confirmed through MALDI-TOF MS or molecular diagnostic methods, an alert mechanism must be immediately activated to notify the infection control team and relevant clinical departments.

### 3.2 Contact precautions and hand hygiene

For confirmed or colonized patients, contact transmission-based precaution measures must be strictly implemented. Patients should be placed in single rooms or cohorted with other patients similarly colonized with *C. auris (*Tsay et al., 2018; Ong et al., 2019; Aldejohann et al., 2022). Healthcare personnel entering the room must wear personal protective equipment, including gloves and gowns, and correctly doff them before exiting (Rapti et al., 2023). All medical equipment brought into the room, such as blood pressure monitors, thermometers, and stethoscopes, should be dedicated whenever possible or thoroughly cleaned and disinfected after use. Reusable invasive equipment, such as video laryngoscope blades, has been identified as a potential transmission vehicle and must undergo strict high-level disinfection or sterilization (Hinrichs et al., 2022).

Strict hand hygiene compliance is the most fundamental and effective measure for interrupting contact transmission. Alcohol-based hand rubs recommended by the World Health Organization should be used for hand hygiene; when hands are visibly soiled, washing with soap and water is required (Ku et al., 2018; Aldejohann et al., 2022; Jones et al., 2024). Notably, the susceptibility of *C. auris* to alcohol-based hand rubs has been confirmed by multiple studies, and conventional concentrations of alcohol-based hand rubs are effective against it (Ku et al., 2018; Rutala et al., 2019). However, sustained hand hygiene compliance remains one of the greatest practical challenges, requiring continuous monitoring, feedback, and training to ensure (Jones et al., 2024).

### 3.3 Environmental cleaning and disinfection

Environmental cleaning and disinfection are paramount in addressing the persistence challenges posed by *C. auris*. Extensive studies have demonstrated that *C. auris* can survive on dry hospital environmental surfaces for weeks or even longer and may exhibit tolerance to certain commonly used disinfectants (Ku et al., 2018; Rutala et al., 2019). Biofilms formed by *C. auris* significantly enhance its resistance to disinfectants and antifungal agents, making contaminated surfaces and equipment more difficult to thoroughly decontaminate; comparative studies of fungal cell wall architecture further elucidate the structural basis for this environmental resilience (Bandara and Samaranayake, 2022).

Multiple studies and reviews have indicated that sodium hypochlorite (bleach)-based disinfectants at appropriate concentrations (e.g., 1000 ppm) demonstrate reliable and consistent efficacy against *C. auris (*Ku et al., 2018; Bandara and Samaranayake, 2022). Other disinfectants, such as hydrogen peroxide, peracetic acid, and certain quaternary ammonium compound formulations, may vary in efficacy depending on product formulation, concentration, and environmental organic load, requiring careful selection based on product specifications and validation data (Rutala et al., 2019). Rutala and colleagues systematically evaluated the efficacy of 21 commonly used healthcare disinfectants against *C. auris* and *C. albicans*, providing an important reference for disinfectant selection (Rutala et al., 2019).

Cleaning procedures should precede disinfection to remove organic material and biofilm matrix (Ku et al., 2018). During outbreaks, the use of vaporized hydrogen peroxide technology for terminal room disinfection may even be warranted (Schelenz et al., 2016; Bandara and Samaranayake, 2022). Polyhexamethylene biguanide hydrochloride has been shown to effectively kill *C. auris* at concentrations far below those commonly used clinically, and ultraviolet-C irradiation can also be employed for environmental decontamination. Furthermore, establishing standardized environmental sampling and monitoring protocols is of significant importance for evaluating cleaning and disinfection efficacy and promptly detecting environmental contamination (Ku et al., 2018).

### 3.4 Inter-facility transfer and regional coordination

Patient transfers between healthcare facilities represent a significant risk point for inter-facility *C. auris* transmission. To ensure safe patient handoffs and prevent outbreak spread, the transferring facility must provide advance notification to the receiving facility regarding the patient’s *C. auris* colonization or infection status (Karmarkar et al., 2021; Jones et al., 2024). The receiving facility should accordingly prepare isolation rooms in advance and arrange for trained healthcare personnel to receive the patient. This information-sharing mechanism is critical for connecting acute care hospitals, long-term care facilities, and rehabilitation institutions within healthcare networks, effectively preventing the silent transmission of the pathogen within the healthcare ecosystem (Karmarkar et al., 2021).

A rapid assessment and containment effort in Orange County, California, USA, provides a successful example. Karmarkar and colleagues reported on the experience of rapid assessment and containment of *C. auris* transmission in post-acute care facilities, emphasizing the importance of inter-facility collaboration, active screening, and timely isolation (Karmarkar et al., 2021). This study demonstrated that implementing coordinated screening and isolation strategies within healthcare facility networks can effectively interrupt *C. auris* transmission chains between acute care hospitals and long-term care facilities.

### 3.5 Experiences and evidence of successful control

Despite the formidable challenges, international experience has demonstrated that through resolute and sustained implementation of comprehensive infection control strategies, *C. auris* transmission can be successfully contained or even eliminated. Outbreak control reports from the UK (Schelenz et al., 2016), Germany (Hinrichs et al., 2022), Kenya (Ombajo et al., 2024), United States (Baker et al., 2025), and Italy (Giovagnorio et al., 2024) provide compelling evidence.

The successful control experience in a COVID-19 ICU in Germany is particularly noteworthy. Hinrichs and colleagues reported that through rapid activation of a multidisciplinary response, extensive screening of high-risk patients, strict implementation of contact precautions, enhanced environmental cleaning and disinfection, and continuous monitoring and feedback on the execution of all measures, *C. auris* transmission was successfully contained (Hinrichs et al., 2022). The control of the first hospital outbreak in the UK also demonstrated the critical role of early identification and rapid response (Schelenz et al., 2016). The successful control measures at a hospital in northern Italy similarly underscored the importance of comprehensive strategies (Giovagnorio et al., 2024). The investigation and successful control experience at a tertiary care facility in Kenya demonstrated that even in resource-limited settings, resolute implementation of basic infection control measures can achieve significant outcomes (Ombajo et al., 2024). Similarly, a study conducted in a Turkish ICU demonstrated that implementing an infection control bundle and checklist resulted in significant reductions in both *C. auris* colonization rates and candidemia incidence (Solak Grassie et al., 2025). Based on the evidence reviewed, we propose a tiered infection prevention and control framework summarized in **Table 1**, categorizing interventions by evidence strength and recommended implementation priority.

**Table 1:** Evidence-based tiered infection prevention and control framework for *Candida auris*.

Tier	Intervention domain	Key components	Evidence level (GRADE)	References
1 (Core)	Multidisciplinary response team	Rapid activation of a multidisciplinary response including clinical specialists of the affected unit, nursing staff, hospital hygiene, diagnostic microbiology, cleaning staff, and hospital management; outbreak preparedness and rapid contact tracing	Strong (High–Moderate)	(Aldejohann et al., 2022; Jones et al., 2024)
	Contact precautions	Single-room isolation or cohorting of colonised/infected patients; single-patient use equipment wherever possible; contact transmission-based precautions for all patient interactions	Strong (High–Moderate)	(Tsay et al., 2018; Ong et al., 2019; Aldejohann et al., 2022)
	Hand hygiene	WHO-recommended alcohol-based hand rubs before and after patient contact; compliance monitoring and feedback; soap and water when hands visibly soiled	Strong (High–Moderate)	(Ku et al., 2018; Aldejohann et al., 2022; Jones et al., 2024)
2 (Enhanced)	Environmental cleaning and disinfection	Daily cleaning with chlorine-based disinfectants (Public Health England (PHE) recommends sodium hypochlorite at 1000 ppm (Ku et al., 2018)); terminal disinfection with hydrogen peroxide vapor when feasible (Ku et al., 2018); cleaning before disinfection to remove organic load	Moderate (Low–Moderate)	(Ku et al., 2018; Rutala et al., 2019; Bandara and Samaranayake, 2022)
	Active surveillance screening	Axilla and groin composite swab screening of high-risk patients on admission and at regular intervals; screening of patients transferred from endemic facilities or with prior *C. auris* exposure	Moderate (Low–Moderate)	(Jones et al., 2024; Baker et al., 2025; Cornely et al., 2025)
3 (Adjunctive)	Antifungal stewardship	Echinocandin as recommended first-line therapy; prospective audit and feedback on antifungal prescriptions; appropriate duration and de-escalation based on susceptibility testing	Moderate (Low–Moderate)	(Arendrup and Patterson, 2017; Eix and Nett, 2025)
	Decolonisation	Chlorhexidine gluconate bathing in outbreak settings, although evidence specific to *C. auris* is limited; octenidine-based wash products showed higher *in vitro* efficacy than CHG; no standardised decolonisation protocol established; effect on human skin not yet studied	Limited (Very Low)	(Gugsch et al., 2024; Jones et al., 2024; Frías-De-León et al., 2025)

Evidence grading adapted from the GRADE system (Guyatt GH, Oxman AD, Vist GE, et al. GRADE: an emerging consensus on rating quality of evidence and strength of recommendations. BMJ 2008;336:924-926). Strong = high-to-moderate quality evidence with strong recommendation supported by multiple national guidelines; Moderate = low-to-moderate quality evidence with conditional recommendation supported by observational studies and expert consensus; Limited = very low quality evidence (no *C. auris*-specific RCTs) with conditional recommendation based on limited data. Tier 1 (Core) = essential measures recommended in all settings; Tier 2 (Enhanced) = measures recommended during active outbreaks; Tier 3 (Adjunctive) = measures with insufficient evidence for routine recommendation.

### 3.6 Antifungal stewardship

Antifungal susceptibility testing (AFST) should be considered an integral component of the routine management of *C. auris* infections and a cornerstone of antifungal stewardship programs. AFST should be performed for all clinical isolates whenever feasible.

Antifungal stewardship programs constitute an essential component of the comprehensive response to *C. auris*, aiming to optimize antifungal use, reduce selective pressure for resistance development, and improve patient outcomes (Arendrup and Patterson, 2017). International guidelines and expert consensus statements emphasize the importance of antifungal stewardship programs that are particularly relevant for *C. auris* management (Arendrup and Patterson, 2017). Key stewardship interventions include prospective audit and feedback on antifungal prescriptions, formulary restriction of broad-spectrum antifungal agents, rapid de-escalation from empirical to targeted therapy based on susceptibility testing results, and appropriate duration of therapy (Arendrup and Patterson, 2017). For *C. auris* specifically, stewardship efforts should prioritize appropriate echinocandin use as first-line therapy while avoiding unnecessary fluconazole exposure that may select for resistance, and ensuring timely escalation to combination therapy or novel agents when clinical response is inadequate (Arendrup and Patterson, 2017). A particularly important stewardship consideration is the appropriate use of novel antifungal agents such as ibrexafungerp and fosmanogepix. While these agents offer valuable therapeutic options for resistant *C. auris* infections, their indiscriminate use could accelerate the emergence of resistance to these last-resort drugs. Stewardship programs should therefore establish clear criteria for novel agent deployment, reserving them for confirmed resistant infections or treatment failures with conventional agents (Arendrup and Patterson, 2017). Integration of stewardship principles with infection control measures creates a synergistic effect: reducing antifungal selection pressure decreases the emergence and spread of resistant strains, while robust infection control limits transmission of existing resistant organisms. Healthcare facilities should establish multidisciplinary antifungal stewardship teams including infectious disease physicians, clinical microbiologists, and pharmacists, with regular review of local *C. auris* epidemiology and resistance patterns to inform institutional treatment guidelines (Arendrup and Patterson, 2017).

### 3.7 Decolonization strategies

Skin decolonization represents a potential adjunctive strategy for reducing *C. auris* transmission, although current evidence remains limited and no standardized protocol has been established. Chlorhexidine gluconate (CHG) bathing has been employed in some outbreak settings, with observational studies suggesting a reduction in skin colonization burden, though complete eradication is rarely achieved and colonization frequently recurs after discontinuation (Gugsch et al., 2024; Jones et al., 2024). A comparative *in vitro* study demonstrated that both chlorhexidine- and octenidine-based wash products exhibited yeasticidal activity against *C. auris*, with octenidine showing higher *in vitro* yeasticidal activity than chlorhexidine in a single comparative study (Gugsch et al., 2024). However, the optimal agent, concentration, frequency, and duration of decolonization remain undefined, and no randomized controlled trial has established the clinical efficacy of any decolonization protocol for *C. auris*. Other investigational approaches include the use of polyhexanide-based antiseptic skin solutions, which have shown promise in reducing *C. auris* colonization and potentially preventing progression to invasive fungemia in colonized patients. Additionally, povidone-iodine-based products have demonstrated *in vitro* activity against *C. auris*, though clinical data are sparse (Frías-De-León et al., 2025). Given these limitations, decolonization should be considered as a supplementary measure alongside standard infection control practices rather than a standalone intervention. It may be most beneficial in outbreak settings where persistent skin colonization contributes to ongoing transmission despite adherence to contact precautions and environmental disinfection. Further research is urgently needed to establish evidence-based decolonization protocols, ideally through well-designed randomized controlled trials comparing different agents and regimens (Frías-De-León et al., 2025).

## 4 Global epidemiological characteristics and transmission dynamics

### 4.1 Geographic distribution and clade characteristics

The global emergence of *C. auris* exhibits a remarkably unique feature: independent, roughly concurrent appearances across multiple geographic locations. Whole-genome sequencing analyses have revealed that the currently circulating *C. auris* strains did not originate from the expansion of a single clone, but rather evolved independently in at least five genetically distinct clades across different geographic regions, with tens of thousands of single nucleotide polymorphism differences between clades (Lockhart et al., 2017; Du et al., 2020). The currently confirmed clades include the South Asian clade (Clade I), East Asian clade (Clade II), African clade (Clade III), South American clade (Clade IV), and Iranian clade (Clade V). In 2024, strains significantly divergent from all known clades were identified in Singapore and Bangladesh, proposed as a novel sixth clade (Clade VI), further revealing the previously unrecognized genetic diversity of this pathogen (Suphavilai et al., 2024).

The geographic distribution and epidemiological characteristics of each clade differ substantially. The South Asian clade (Clade I) is currently the most widely disseminated and most resistant clade, responsible for large-scale nosocomial outbreaks in India, Pakistan, the United Kingdom, the United States, Greece, and numerous other countries (Chow et al., 2020; Meletiadis et al., 2024). In the United States, since its first report in 2016, *C. auris* case numbers have risen sharply, with clinical cases increasing by 95% in 2021 compared to the previous year, and colonization cases identified through screening increasing by over 200% (Lyman et al., 2023). Surveillance data indicate that by 2022, *C. auris* accounted for 1.6% of invasive candidiasis isolates, compared to less than 0.1% prior to 2018 (Castanheira et al., 2024). The South American clade (Clade IV), initially identified in Venezuela, has subsequently spread widely throughout Colombia, Panama, and other Latin American countries, causing sustained endemic transmission (Escandon, 2022; Misas et al., 2024). National laboratory surveillance in Colombia documented 1,720 *C. auris* cases between 2016 and 2020, affecting 62% of the country’s departments (Escandon, 2022). The East Asian clade (Clade II) is predominantly found in Japan and Korea, characterized by relatively lower fluconazole resistance (31%) compared to Clades I and III, though notable micafungin resistance (30%, n=27) has been observed; Japanese isolates are primarily derived from ear discharge specimens and are generally susceptible to amphotericin B and anidulafungin (Iguchi et al., 2019; Abe et al., 2025). The African clade (Clade III) was initially discovered in South Africa and subsequently spread to the Middle East and Europe (Spettel et al., 2023).

With the strengthening of global surveillance networks, an increasing number of countries and regions have reported their first *C. auris* cases, including Austria (Spettel et al., 2023), Russia (Barantsevich et al., 2019), Taiwan (Tsai et al., 2022), and Germany (Hinrichs et al., 2022). In mainland China, systematic analyses have shown that the isolation rate of *C. auris* remains relatively low, but all tested isolates were resistant to fluconazole (Bilal et al., 2022). Collectively, these reports depict the dynamic landscape of *C. auris* continuing to spread through hospital networks and international travel.

### 4.2 Transmission patterns within healthcare facilities

Healthcare facilities, particularly intensive care units (ICUs) and skilled nursing facilities, constitute the primary settings for *C. auris* transmission and infection outbreaks (Yune et al., 2023; Eix and Nett, 2025). Unlike other *Candida* species that predominantly cause endogenous infections, *C. auris* possesses robust skin colonization capacity, persisting in patient axillae, groin, and other body sites, and transmitting between patients through direct contact or contaminated medical equipment and environmental surfaces (Eix and Nett, 2025). Its ability to survive on dry surfaces for weeks and tolerate commonly used hospital disinfectants poses formidable challenges for environmental cleaning and decontamination (Yune et al., 2023).

Skin colonization is the cornerstone of *C. auris* nosocomial transmission. Studies have demonstrated that *C. auris* preferentially colonizes the hair follicle niche, effectively binding to human hair and inducing a local interferon-gamma-dominated type 1 immune response; this response, rather than clearing the fungus, paradoxically compromises epithelial barrier integrity, thereby creating a favorable microenvironment for persistent colonization (Briano et al., 2022). Colonization is not only a risk factor for subsequent invasive infection but also serves as the primary source of inter-personal transmission and environmental contamination within hospitals (Chowdhary et al., 2025). A study conducted in an ICU demonstrated that *C. auris*-colonized patients face a substantial risk of subsequent bloodstream infection, with multi-site colonization being an independent risk factor for candidemia and a cumulative incidence exceeding 25% within 60 days of colonization (Kappel et al., 2024).

Outbreaks are frequently associated with invasive procedures (such as central venous catheter placement), broad-spectrum antibiotic and antifungal agent use, and severe underlying patient conditions (Yune et al., 2023; Eix and Nett, 2025). Molecular epidemiological studies have played a pivotal role in tracing nosocomial transmission. Analyses of isolates from multiple UK hospitals not only identified independent international importation events but also revealed clear transmission chains of resistant strains both within and between hospitals (Kean and Ramage, 2019). During a nearly three-year outbreak at a Greek tertiary care hospital, all bloodstream infection isolates belonged to Clade I and were genetically related, with infection peaks closely correlating with colonization rate peaks, ultimately resulting in *C. auris* becoming the predominant cause of candidemia at that institution (34%) in 2023 (Meletiadis et al., 2024). Biofilm formation further enhances the persistence of *C. auris* in healthcare environments, and strains isolated during outbreaks typically exhibit high biofilm-forming capacity (Khojasteh et al., 2022).

### 4.3 Impact of the COVID-19 pandemic on epidemiological patterns

The COVID-19 pandemic profoundly altered the epidemiological landscape of *C. auris*. COVID-19 patients, particularly those requiring mechanical ventilation and ICU admission, represent an extremely high-risk population for *C. auris* colonization and infection due to immunosuppressive states (such as corticosteroid use), broad-spectrum antibiotic therapy, invasive procedures, and the potential compromises in infection control practices secondary to healthcare system strain and staffing shortages (Khojasteh et al., 2022; Najeeb et al., 2022).

Beyond the COVID-19 pandemic, the broader principle that healthcare system disruptions can facilitate the emergence and spread of *C. auris* is supported by evidence from other crises. During the 2014–2016 Ebola epidemic in West Africa, the near-complete diversion of healthcare resources toward outbreak response led to the disruption of routine infection prevention and control practices. These observations underscore a critical insight: the epidemiological vulnerability to *C. auris* is not specific to COVID-19 but rather reflects a broader pattern in which any event that substantially strains healthcare infrastructure can create conditions favorable for the emergence and spread of resistant fungal pathogens.

Multiple countries have reported significant increases in *C. auris* infection rates during the COVID-19 pandemic. In Israel, the incidence of *C. auris* surged 30-fold in 2021, temporally coinciding with COVID-19 hospitalization peaks; the outbreak initially involved dedicated COVID-19 wards before spreading to non-COVID-19 mechanically ventilated patients and non-ventilated patients (Biran et al., 2023). During the COVID-19 era, the overall incidence of candidemia increased in several countries, with the proportion attributable to *C. auris* rising significantly. Similarly, 30% of cases reported in Colombia in 2020 were associated with severe COVID-19 (Escandon, 2022). A study from Qatar noted that the majority of patients with *C. auris* bloodstream infections were admitted to the ICU for severe COVID-19 pneumonia and had received corticosteroids and broad-spectrum antibiotics (Maphanga et al., 2021). However, research from Hong Kong, China, indicated that the emergence of *C. auris* was not directly associated with changes in antifungal prescription volumes, underscoring the central role of proactive infection control measures in preventing nosocomial transmission (Sharma et al., 2022).

### 4.4 Environmental reservoirs and clues of community transmission

A growing body of evidence suggests that climate change and global warming may play a significant role in the emergence and adaptation of *C. auris* as a human pathogen. Casadevall and colleagues have proposed that increasing global temperatures are exerting selective pressure on environmental fungi, driving their thermal adaptation toward mammalian body temperatures (Casadevall et al., 2019). *C. auris* is notably thermotolerant, capable of growing at 42 °C. The simultaneous emergence of genetically distinct *C. auris* clades on three continents has been hypothesized to be consistent with a climate-driven emergence event (Casadevall et al., 2019). Supporting the environmental reservoir hypothesis, *C. auris* and closely related species have been isolated from natural habitats, including coastal wetlands and salt marshes of the Andaman Islands (Arora et al., 2021).

The ecological origins of *C. auris* have traditionally been considered unknown, but recent studies have provided novel insights. Researchers have isolated genetically diverse *C. auris* Clade I strains from the surfaces of stored apples, which were closely related to strains from Indian patients, hospitals, and marine environments (Yadav et al., 2022). Apples during storage are commonly treated with fungicides including triazoles, and all isolates exhibited reduced triazole susceptibility, suggesting that agricultural fungicide use may be an important driver selecting for azole resistance in natural environments (Yadav et al., 2022).

Wastewater-based epidemiology, as an emerging surveillance tool, has demonstrated potential for early warning of *C. auris* transmission in communities or healthcare facilities. In studies from the United States, *C. auris* was consistently detected from wastewater catchments serving healthcare facilities with known outbreaks, with detection rates significantly higher than those from catchments without outbreak facilities (Barber et al., 2023). These findings suggest that *C. auris* may exist in broader environmental niches, and its transmission dynamics may be more complex than currently appreciated based on clinical case surveillance alone. Importantly, while environmental reservoirs and wastewater detection provide valuable early warning signals, the relative contribution of environmental contamination versus direct patient-to-patient transmission to overall *C. auris* spread remains poorly quantified, representing a critical gap in the current epidemiological evidence base.

## 5 Antifungal resistance epidemiology

### 5.1 Global distribution of resistance profiles

Antifungal resistance is a key factor enabling *C. auris* to cause nosocomial outbreaks and contribute to high mortality rates (**Table 2**). Its resistance profiles exhibit pronounced geographic variation and clade specificity, which directly influence clinical treatment choices and infection control strategies across different regions.

**Table 2:** Antifungal resistance profiles of *Candida auris* by clade.

Clade	Geographic origin	Fluconazole resistance	Amphotericin B resistance	Echinocandin resistance (AFG/CAS/MCF)	Key resistance mechanisms
I (South Asian)	India/Pakistan	94%	46%	3.7%/22.6%/<5%	*ERG11* Y132F/K143R; *FKS1* S639P
II (East Asian)	Japan/S. Korea	31%	No resistance	No resistance/5.3%/30%	No*ERG11* mutations; MCF resistance notable
III (African)	South Africa	96%	6%	0.75%/1.65%/<5%	*ERG11* F126L; *FKS1* S639F
IV (S. American)	Venezuela/Colombia	44%	22%	1.0%/2.9%/6.1%	*ERG11* Y132F; *FKS1* S639P
V (Iranian)	Iran	Resistant (n=7; see text)	No resistance	No resistance/No resistance/No resistance	Under investigation
VI (Proposed)	Singapore	No resistance (n=4)	33% (1/3)	No resistance/No resistance/No resistance	Under investigation

Echinocandin resistance is reported separately for anidulafungin (AFG), caspofungin (CAS), and micafungin (MCF) due to significant inter-drug variability. All resistance data from da Silva et al. (Clin Microbiol Infect 2025). Clade V (n=7) and Clade VI (n=4) data should be interpreted with caution due to extremely limited isolate numbers.

Regarding azole resistance, the vast majority of *C. auris* clinical isolates are resistant to fluconazole, with resistance rates of 94% in Clade I and 96% in Clade III, while Clade IV shows 44% fluconazole resistance. Clades I and III constitute the majority of clinical isolates globally (Chowdhary et al., 2023; Kim et al., 2024). The azole resistance mechanisms differ among clades and involve both target gene mutations and efflux pump overexpression, as detailed in Section 5.4 (AlJindan et al., 2020; Chow et al., 2020; Maphanga et al., 2021). The East Asian clade (Clade II) is an exception, with relatively low azole resistance rates (Iguchi et al., 2019; Abe et al., 2025).

Regarding echinocandin resistance, this drug class is currently widely recommended as first-line therapy for invasive infections. However, the emergence and spread of echinocandin resistance represents an increasingly grave threat. Such resistance is primarily mediated by *FKS1* hotspot mutations (detailed in Section 5.4), resulting in cross-resistance to all echinocandin agents (Kordalewska et al., 2018; Sharma et al., 2022). Pan-echinocandin-resistant isolates have even emerged during an outbreak at a Greek tertiary care hospital (Meletiadis et al., 2024). Both clinical and *in vivo* studies have confirmed that *FKS1* mutations are the most reliable molecular markers for predicting echinocandin clinical resistance in *C. auris (*Sharma et al., 2022).

Resistance to polyene agents (such as amphotericin B) varies considerably across clades: Clade I shows the highest amphotericin B resistance rate at 46%, Clade IV at 22%, Clade III at only 6%, and Clades II and V are fully susceptible (da Silva et al., 2025). Notably, certain commercial susceptibility testing kits may overestimate amphotericin B resistance rates, necessitating method-specific breakpoints for accurate interpretation (Wang et al., 2025). Of particular concern, pan-resistant strains demonstrating resistance to all three major classes of antifungal agents (azoles, echinocandins, and polyenes) have emerged (Jacobs et al., 2022), leaving extremely limited therapeutic options.

### 5.2 Association between resistant clades and transmission

A close bidirectional association exists between antifungal resistance and transmission dynamics in *C. auris*. On one hand, high resistance rates are an important driver of transmission success–under antifungal selection pressure, resistant strains possess a survival advantage over susceptible strains, enabling persistent colonization and transmission in patients receiving prophylactic or therapeutic antifungal agents (Arendrup and Patterson, 2017). On the other hand, transmission itself further disseminates resistance, particularly when resistant strains establish clonal transmission within healthcare facilities (Spruijtenburg et al., 2023; Kappel et al., 2024). Based on the evidence reviewed, the resistance-transmission nexus appears to represent the most critical intersection for *C. auris* control: interrupting transmission reduces the patient-days of antifungal exposure that drive resistance, while containing resistance preserves therapeutic options that reduce transmission-driven mortality.

Whole-genome sequencing studies have demonstrated that the major globally circulating *C. auris* clades emerged independently and nearly simultaneously from different geographic regions within recent decades (Lockhart et al., 2017; Chow et al., 2020; Du et al., 2020). This unique evolutionary history implies that each clade has faced distinct local selection pressures, thereby shaping their unique resistance profiles. For example, the high fluconazole resistance rate in Clade I in South Asia may be associated with the extensive use of fluconazole for empirical therapy in that region, whereas the lower resistance rate in Clade II in East Asia may reflect different antifungal usage patterns (Iguchi et al., 2019; Abe et al., 2025).

In clinical settings, longitudinal genomic analyses of serial isolates from the same patient have directly observed *C. auris* evolving from resistance to a single drug class to resistance to multiple classes through the acquisition of novel mutations in *ERG11*, *FKS1*, and other genes during antifungal therapy (Spruijtenburg et al., 2023; Wang et al., 2025). This rapid *in vivo* resistance evolution means that even initially susceptible infecting strains may develop resistance during treatment, thereby increasing the risk of treatment failure and sustained transmission. *In vitro* experimental evolution studies have also clearly demonstrated that *C. auris* can rapidly evolve multidrug resistance through the stepwise accumulation of chromosomal copy number variations and target gene mutations under antifungal pressure (Carolus et al., 2021).

### 5.3 Biofilm formation and antifungal tolerance

Biofilm formation represents a critical virulence factor and a major driver of *C. auris* persistence in healthcare environments. Biofilm-associated *C. auris* cells (sessile cells) exhibit substantially reduced susceptibility to antifungal agents, with MBECs ranging from 2- to 4,119-fold higher than planktonic MICs (Ceballos-Garzon et al., 2025). The mechanisms include: (i) restricted drug penetration through the extracellular matrix (ECM); (ii) upregulation of efflux pumps, including ABC transporters (*CDR1*) and MFS transporters (*MDR1*) (Kean and Ramage, 2019; Ceballos-Garzon et al., 2025); (iii) a subpopulation of persister cells that may survive antifungal exposure in a metabolically dormant state (Ceballos-Garzon et al., 2025); and (iv) alterations in cell wall composition, including increased mannoproteins and reduced beta-glucan exposure (Chatzimoschou et al., 2021; Ceballos-Garzon et al., 2025). Clinically, *C. auris* biofilms have been implicated in persistent colonization of medical devices and extensive surface contamination in healthcare environments (Kean and Ramage, 2019). Importantly, the antibiofilm activity of both conventional and novel antifungal agents has been evaluated against *C. auris* biofilms. A comprehensive study using the Calgary biofilm device assessed five antifungal agents against both planktonic cells and mature biofilms (Ceballos-Garzon et al., 2025). Among these, manogepix (the active moiety of fosmanogepix) demonstrated the highest overall antibiofilm activity (geometric mean MBEC of 5.9 μg/mL), while ibrexafungerp showed superior activity against Clade IV biofilms (Ceballos-Garzon et al., 2025). The marked discrepancy between planktonic MICs and biofilm MBECs (up to 4,119-fold) underscores the considerable therapeutic challenge posed by *C. auris* biofilms and highlights the importance of evaluating antifungal efficacy against both planktonic and sessile cells.

### 5.4 Resistance mechanisms

Understanding the molecular mechanisms underlying *C. auris* antifungal resistance is essential for guiding therapeutic decisions and developing novel treatment strategies. The resistance mechanisms in *C. auris* are multifactorial and involve target gene mutations, overexpression of efflux pumps, and biofilm-associated persistence (Arendrup and Patterson, 2017; Chowdhary et al., 2025). Azole resistance in *C. auris* is primarily mediated by mutations in the *ERG11* gene encoding lanosterol 14-alpha-demethylase, the target enzyme of azole antifungals. Three hotspot mutations have been identified with distinct clade associations: Y132F and K143R substitutions are commonly observed in Clade I (South Asian) and Clade IV (South American) isolates, while F126L is characteristic of Clade III (African) isolates (Arendrup and Patterson, 2017; Chowdhary et al., 2025). These mutations alter the azole-binding site, substantially reducing drug affinity. Additionally, overexpression of *ERG11* due to promoter upregulation further contributes to azole resistance by increasing the amount of target enzyme that must be inhibited (Chowdhary et al., 2025). Importantly, overexpression of ATP-binding cassette (ABC) transporter efflux pumps, particularly *CDR1*, and major facilitator superfamily (MFS) transporters, such as *MDR1*, represents a predominant mechanism of azole resistance in *C. auris*. Gain-of-function mutations in the transcription factors *Tac1b* and *Mrr1*, which regulate *CDR1* and *MDR1* expression respectively, drive constitutive overexpression of these efflux pumps, effectively reducing intracellular drug accumulation (Chowdhary et al., 2025).

Echinocandin resistance arises from mutations in the *FKS1* gene encoding beta-1,3-glucan synthase, the target of echinocandin drugs. The most commonly observed hotspot mutations include S639F/P/Y in the conserved region of *FKS1*, which reduce echinocandin binding affinity and elevate MIC values by 10- to 100-fold (Arendrup and Patterson, 2017; Chowdhary et al., 2025). Unlike azole resistance, echinocandin resistance in *C. auris* is predominantly acquired during patient treatment rather than being clade-intrinsic, as evidenced by serial isolate analyses demonstrating *de novo FKS1* mutation emergence under echinocandin selective pressure (Arendrup and Patterson, 2017).

Amphotericin B resistance mechanisms in *C. auris* are less well characterized. In *Candida* species generally, combined mutations in *ERG11* and *ERG2*, have been associated with depletion of ergosterol and amphotericin B resistance (Arendrup and Patterson, 2017). In *C. auris* specifically, a mutation in the sterol-methyltransferase gene *ERG6* was recently linked to increased amphotericin B resistance (Chowdhary et al., 2025). Some studies have also implicated increased oxidative stress response pathways in amphotericin B tolerance. Notably, certain commercial susceptibility testing methods, particularly the Sensititre YeastOne system, may overestimate amphotericin B resistance rates, and broth microdilution remains the reference standard for accurate determination (Siopi et al., 2023). Of particular concern, *C. auris* can acquire resistance to multiple drug classes simultaneously through the accumulation of independent mutations in *ERG11*, *FKS1*, and other resistance genes during prolonged antifungal exposure, ultimately leading to pan-resistant isolates that are refractory to all clinically available antifungal agents (Arendrup and Patterson, 2017; Chowdhary et al., 2025).

### 5.5 Current treatment recommendations

The emergence of pan-resistant *C. auris* strains has urgently driven the development of novel antifungal agents. Ibrexafungerp (formerly SCY-078), an oral glucan synthase inhibitor belonging to the triterpenoid class, has demonstrated potent *in vitro* activity against *C. auris* including echinocandin-resistant strains harboring *FKS1* mutations, with modal minimum inhibitory concentrations (MICs)/MIC50 of 0.5 mg/L by European Committee on Antimicrobial Susceptibility Testing (EUCAST) broth microdilution (Chowdhary et al., 2025). It received FDA approval in June 2021 for vulvovaginal candidiasis and represents the first new antifungal class approved in over two decades. Its oral bioavailability offers a significant advantage for step-down therapy and outpatient management of *C. auris* infections, although clinical data specifically for invasive *C. auris* candidemia remain limited to case reports and compassionate use (Chowdhary et al., 2025). Fosmanogepix (formerly APX001), a prodrug of the Gwt1 inhibitor apx001, targets fungal glycosylphosphatidylinositol anchor biosynthesis and has shown promising *in vitro* activity against all five *C. auris* clades, including strains resistant to azoles, echinocandins, and amphotericin B (Chowdhary et al., 2025). A phase 2 clinical trial demonstrated an 89% survival rate in patients with invasive candidemia caused by *C. auris*, and a phase 3 trial (ReVITAL) is currently ongoing (Chowdhary et al., 2025). Its novel mechanism of action avoids cross-resistance with existing antifungal classes, making it particularly valuable for managing multidrug-resistant infections. Rezafungin, a next-generation echinocandin with an extended half-life of approximately 70 hours enabling once-weekly intravenous dosing, received FDA approval in 2023 for the treatment of candidemia and invasive candidiasis (Chowdhary et al., 2025). This dosing schedule could improve adherence in long-term care settings and facilitate outpatient antifungal therapy (Chowdhary et al., 2025). Collectively, these novel agents represent critically important therapeutic alternatives for managing infections caused by multidrug-resistant and pan-resistant *C. auris*. However, robust clinical outcome data from randomized controlled trials involving *C. auris* patients are still needed, and their optimal positioning within treatment algorithms warrants further investigation. It is important to note that the biofilm activity of these agents differs from their planktonic activity: as detailed in Section 5.3, manogepix demonstrates the highest antibiofilm activity (geometric mean MBEC of 5.9 μg/mL), while ibrexafungerp shows superior activity against Clade IV biofilms (Ceballos-Garzon et al., 2025). These biofilm data underscore the need for agents that are effective against both planktonic and sessile *C. auris* cells, as infections associated with biofilm formation on medical devices represent a particularly challenging clinical scenario. In addition to novel single-agent therapies, combination antifungal regimens have been explored as a strategy to enhance efficacy and prevent the emergence of resistance in *C. auris* infections. *In vitro* studies have reported synergistic interactions between amphotericin B and echinocandins against *C. auris* planktonic cells, with fractional inhibitory concentration index (FICI) values ranging from 0.076 to 0.5 indicating synergy in most combinations; the combination of amphotericin B and caspofungin showed the strongest protective effect, achieving up to 99% survival in the *Caenorhabditis elegans* host model (Arendrup and Patterson, 2017; Chowdhary et al., 2025). The combination of echinocandins with azoles has also shown additive to synergistic effects in some studies, although the clinical significance of these *in vitro* findings remains to be validated in prospective clinical trials (Arendrup and Patterson, 2017). Triple-combination approaches incorporating novel agents such as ibrexafungerp with existing antifungals represent an area of active investigation (Chowdhary et al., 2025). Current clinical practice generally reserves combination therapy for patients failing or intolerant to first-line echinocandin treatment, rather than as initial empirical therapy (Arendrup and Patterson, 2017; Chowdhary et al., 2025). The optimal combination regimens, treatment durations, and patient selection criteria remain important unresolved questions that require well-designed clinical studies to address.

The management of *C. auris* infections requires a structured, evidence-informed approach that accounts for the pathogen’s multidrug resistance profile and the limited availability of clinical outcome data. Current treatment recommendations are primarily derived from observational studies, case series, and expert consensus, as no randomized controlled trials have been conducted specifically for *C. auris* candidemia (Arendrup and Patterson, 2017; Chowdhary et al., 2025). First-line therapy for invasive *C. auris* infections consists of an echinocandin agent (anidulafungin, caspofungin, or micafungin), consistent with recommendations from the CDC, European Society of Clinical Microbiology and Infectious Diseases (ESCMID), and Infectious Diseases Society of America (IDSA) for all invasive candidiasis (Arendrup and Patterson, 2017; Chowdhary et al., 2025). This recommendation is supported by the generally low echinocandin resistance rates across most clades, with anidulafungin demonstrating the greatest efficacy (resistance rates 0-3.7% across clades) (da Silva et al., 2025). However, given the rising echinocandin resistance observed in Clade I (caspofungin resistance up to 22.6%), it is advisable that initial echinocandin selection consider local resistance epidemiology, and anidulafungin or micafungin may be preferred over caspofungin in regions where Clade I predominates. The main toxicities and limitations of current antifungal agents warrant consideration in treatment decisions. Amphotericin B, while broadly active, is associated with significant nephrotoxicity and infusion-related reactions, necessitating careful monitoring and premedication (Laniado-Laborín and Cabrales-Vargas, 2009). Lipid formulations reduce nephrotoxicity but remain costly, limiting access in resource-constrained settings (Laniado-Laborín and Cabrales-Vargas, 2009). Echinocandins are generally well tolerated with a favorable safety profile, but require intravenous administration (Cornely et al., 2025). Fluconazole, though convenient for oral step-down therapy, is limited by high resistance rates that must be considered in treatment decisions (Cornely et al., 2025). Among novel agents, ibrexafungerp is associated with gastrointestinal adverse effects including nausea, vomiting, abdominal pain, and diarrhea (El Ayoubi et al., 2024), fosmanogepix has been well tolerated in phase 2 trials but safety data remain limited (Vazquez et al., 2023), and rezafungin has demonstrated a safety profile comparable to caspofungin in phase 2 trials (Thompson et al., 2021); however, data specifically in *C. auris*-infected patients remain limited. When combination therapies are considered, the toxicological burden may be compounded; for example, the inherent nephrotoxicity of amphotericin B (Laniado-Laborín and Cabrales-Vargas, 2009) and the requirement for intravenous access with both amphotericin B and echinocandins present cumulative challenges, while the synergistic benefits observed *in vitro* have not been validated in prospective clinical trials (Arendrup and Patterson, 2017; Chowdhary et al., 2025). These limitations highlight the importance of individualized treatment decisions guided by susceptibility testing, patient comorbidities, and available resources. Step-down therapy should be guided by antifungal susceptibility testing results. For fluconazole-susceptible isolates (primarily from Clades II and V), oral fluconazole offers a convenient step-down option. For fluconazole-resistant isolates, oral ibrexafungerp represents a promising step-down alternative, though clinical data for invasive *C. auris* infections remain limited (Chowdhary et al., 2025). In the authors’ view, based on the available evidence, the decision to step down should balance the convenience of oral therapy against the limited evidence base, and intravenous echinocandin continuation may be preferable for critically ill patients. Combination antifungal therapy should be considered in two scenarios: (i) patients with pan-resistant or XDR isolates for whom no single-agent option retains activity, and (ii) patients with persistent candidemia despite appropriate first-line therapy for >5 days (Arendrup and Patterson, 2017; Chowdhary et al., 2025). The combination of amphotericin B and caspofungin has shown the strongest *in vitro* and *in vivo* synergistic activity (FICI 0.076-0.5; 99% survival in the *Caenorhabditis elegans* host model (Arendrup and Patterson, 2017; Chowdhary et al., 2025)), though clinical validation is lacking. Current expert consensus suggests that combination therapy be reserved as a salvage approach rather than initial empirical therapy, pending results of prospective clinical trials. Special populations require particular attention. In neutropenic patients, the risk of breakthrough candidemia on echinocandin prophylaxis is a concern, and high-dose amphotericin B lipid formulation may be considered as initial therapy. In neonatal ICU settings, where *C. auris* outbreaks have been reported, dosing adjustments and safety profiles of echinocandins require careful consideration. An important unresolved question is whether the novel agents ibrexafungerp, fosmanogepix, and rezafungin will demonstrate clinical efficacy for invasive *C. auris* infections in phase 3 trials, which would substantially expand the therapeutic armamentarium.

## 6 Diagnosis and surveillance

### 6.1 Challenges and advances in species identification

*C. auris* presents unique diagnostic challenges. Historically, this organism has been frequently misidentified (Keighley et al., 2021) as other *Candida* species by conventional biochemical yeast identification systems, particularly members of the *Candida haemulonii* complex (Dewaele et al., 2019; Iguchi et al., 2019). A survey in Belgium revealed that when using conventional workflows, only approximately 57.7% of laboratories could correctly identify *C. auris*, while approximately 19% misidentified it as *C. haemulonii (*Dewaele et al., 2019). Such misidentification directly leads to delayed implementation of infection control measures and constitutes a major early obstacle in combating its spread. The extent of misidentification varies considerably by system: VITEK 2 YST (versions prior to 8.01) frequently misidentifies *C. auris* as *C. haemulonii* or C. duobushaemulonii, with misidentification rates exceeding 50% in some studies; API 20C AUX and API ID 32C similarly fail to differentiate *C. auris* from related species; and BD Phoenix and MicroScan systems lack *C. auris* in their databases entirely, precluding correct identification (Dewaele et al., 2019; Iguchi et al., 2019). These limitations underscore the critical importance of employing updated identification platforms and molecular confirmatory methods for accurate *C. auris* detection.

Database updates for matrix-assisted laser desorption/ionization time-of-flight mass spectrometry (MALDI-TOF MS) have substantially improved identification accuracy (Jamalian et al., 2023). Culture-based chromogenic media (such as CHROMagar Candida Plus) also serve as important foundational tools for laboratory screening, enabling differentiation of *C. auris* from other *Candida* species through the production of specific colony colors (Tamura et al., 2022; Rahman et al., 2024). However, approximately 44.3% of Candida parapsilosis strains may exhibit similar morphology on this medium, causing false positives; some studies have recommended supplementing the medium with fluconazole to improve discriminatory specificity (Ruiz-Gaitan et al., 2023)].

Beyond MALDI-TOF MS and chromogenic media, several advanced molecular diagnostic platforms have been developed or adapted for *C. auris* detection. Real-time polymerase chain reaction (PCR), loop-mediated isothermal amplification (LAMP), and T2 magnetic resonance assays have been explored for *C. auris* identification, though the standard T2Candida panel does not include *C. auris*-specific detection (Chowdhary et al., 2025). Two nucleic acid-based diagnostic assays have recently been approved by the FDA for testing *C. auris*-positive blood culture samples: the GenMark ePlex Blood Culture Identification Fungal Pathogen (BCID-FP) panel and the BioFire FilmArray BCID2 Panel, the latter of which includes *C. auris*-specific probes enabling direct species-level identification (Chowdhary et al., 2025). Additionally, TaqMan chemistry-based probe assays have been developed for rapid detection of echinocandin resistance-conferring *FKS1* mutations in *C. auris (*Jolivet et al., 2025). These molecular platforms significantly reduce the time to appropriate infection control interventions compared to culture-based methods, although their availability and cost may limit deployment in resource-constrained settings.

### 6.2 Active screening and colonization surveillance

Active surveillance screening is a critical component for early case detection and interruption of transmission chains. Given that *C. auris* is primarily transmitted through contact and skin colonization is common, screening should focus on high-risk patient populations (Rapti et al., 2023), including patients transferred from *C. auris*-endemic areas or healthcare facilities with known outbreaks, close contacts of confirmed cases, and patients in high-risk wards such as ICUs, long-term acute care hospitals, and burn units (Kordalewska et al., 2019; Welsh et al., 2021; Mezzogori et al., 2026). Screening sites typically target the axillae and groin, where colonization rates are highest, using composite swab sampling (Mezzogori et al., 2026).

Although conventional culture remains the gold standard, it is time-consuming. Real-time quantitative PCR (qPCR) technology can directly detect *C. auris* DNA from patient swab specimens, reducing detection time from days to hours, which is of paramount importance for the timely implementation of infection control measures (Crawford et al., 2022; Zhu et al., 2023). Multiple studies have confirmed the value of qPCR in screening (Ruiz-Gaitan et al., 2023), demonstrating greater sensitivity than culture methods and enabling earlier identification of colonized patients and environmental contamination, thereby facilitating rapid patient cohorting and environmental decontamination (Aldejohann et al., 2022). Additionally, loop-mediated isothermal amplification (LAMP) technology provides a rapid detection option for resource-limited settings.

### 6.3 Molecular epidemiological typing

Molecular epidemiological typing techniques provide powerful tools for tracing transmission chains and identifying different clades, serving as an important basis for infection control decision-making. Allele-specific PCR based on internal transcribed spacer regions or specific gene clusters enables rapid, low-cost identification of the five major *C. auris* clades (Mezzogori et al., 2026). Whole-genome sequencing (WGS) currently offers the highest resolution typing method, capable of precisely revealing phylogenetic relationships between strains for outbreak investigation and global transmission dynamics research (Keighley et al., 2021; Welsh et al., 2021). To facilitate data comparability across different surveillance networks, standardized WGS benchmark datasets have been established (Welsh et al., 2021).

Several novel rapid typing technologies are also under investigation. Furthermore, qPCR with specific probes can directly detect *ERG11* gene mutations conferring azole resistance from clinical specimens (Kordalewska et al., 2019; Jolivet et al., 2025). This “culture-independent” rapid resistance assessment holds promise for providing critical information to guide antifungal therapy selection and infection control grading at an early stage of patient management. *FKS1* gene hotspot mutations conferring echinocandin resistance and key.

## 7 Conclusions

*Candida auris*, as a nosocomial pathogen combining multidrug resistance, diagnostic difficulty, environmental persistence, and efficient transmission, poses a sustained and formidable challenge to modern medicine (Forsberg et al., 2019; Chowdhary et al., 2023). This review, with epidemiology and infection control as its central thread, has systematically synthesized the latest advances in the global epidemiological characteristics, antifungal resistance epidemiology, diagnostic and surveillance technologies, and infection prevention and control strategies for this pathogen.

In terms of epidemiology, *C. auris* has formed five confirmed and one proposed clade (Clade VI) with significant differences in resistance profiles and transmissibility, and this diversity increases the complexity of prevention and control. Contact transmission within healthcare facilities constitutes the primary route, skin colonization is the cornerstone of transmission, and the COVID-19 pandemic has further exposed healthcare system vulnerabilities in infection control. Novel findings regarding environmental reservoirs and wastewater surveillance suggest that *C. auris* transmission dynamics likely involve pathways not fully captured by clinical case surveillance alone.

In terms of resistance epidemiology, fluconazole resistance rates of 94% in Clade I and 96% in Clade III represent the highest burden, while Clade IV shows 44% resistance. The emergence and spread of echinocandin-resistant strains pose a severe threat to clinical management, and the appearance of pan-resistant strains sounds an even greater alarm. The bidirectional association between resistance and transmission implies that antifungal stewardship is not merely a therapeutic issue but a core component of infection control.

In terms of diagnosis and surveillance, the application of MALDI-TOF MS and molecular detection techniques has substantially improved identification capacity, while active screening and wastewater surveillance have provided novel tools for early warning. However, in resource-limited regions, insufficient diagnostic capacity remains a major barrier to effective surveillance and response.

Based on the evidence reviewed, several research priorities and public health considerations emerge. The expansion of MALDI-TOF MS with updated databases and molecular confirmatory testing across clinical microbiology laboratories represents a foundational need, as accurate identification is the prerequisite for all subsequent interventions. Active surveillance screening of high-risk patient populations upon admission to healthcare facilities, particularly in ICUs and long-term care settings, warrants broader implementation and evaluation. The strengthening of contact precautions and environmental disinfection protocols using chlorine-based agents at validated concentrations remains essential, as does the development of regional and national *C. auris* surveillance networks with real-time data sharing to enable early detection of clonal spread. Looking ahead, the accelerated development and clinical evaluation of novel antifungal agents, particularly ibrexafungerp, fosmanogepix, and rezafungin, is needed to address the growing threat of pan-resistance, and investment in point-of-care diagnostic technologies suitable for resource-limited settings—where the burden of *C. auris* is increasingly recognized but diagnostic capacity remains most constrained (Spettel et al., 2023)—is a critical long-term priority. For resource-limited regions, low-cost interventions merit particular consideration: chromogenic culture media for screening, strict adherence to basic hand hygiene and contact precautions, and inter-facility communication networks using existing public health infrastructure. These measures, while simple, have proven effective in outbreak containment in Kenya and other resource-constrained settings (Ombajo et al., 2024).

## 8 Future perspectives

Several critical knowledge gaps remain: the ecological niche and environmental reservoirs of *C. auris* are poorly defined; the clinical efficacy of novel antifungal agents for invasive *C. auris* infections awaits phase 3 trial results; no randomized controlled trials have evaluated decolonization strategies; and the epidemiological characteristics of the recently identified Clade VI are virtually unknown. Addressing these gaps should be prioritized in future research.

There is no single approach for overcoming the *C. auris* threat; it relies on the continuous strengthening of epidemiological surveillance, rapid iteration of diagnostic technologies, resolute execution of infection control measures, and close collaboration within global public health systems. Only through comprehensive and coordinated efforts can the spread of *C. auris* be mitigated, and preparedness enhanced for confronting future emerging drug-resistant pathogens.
